# Scintigraphic tracking of ^99m^Technetium-labelled equine peripheral blood-derived mesenchymal stem cells after intravenous, intramuscular, and subcutaneous injection in healthy dogs

**DOI:** 10.1186/s13287-021-02457-9

**Published:** 2021-07-13

**Authors:** Charlotte Beerts, Carlien Brondeel, Glenn Pauwelyn, Eva Depuydt, Liesa Tack, Luc Duchateau, Yangfeng Xu, Jimmy H. Saunders, Kathelijne Peremans, Jan H. Spaas

**Affiliations:** 1 Global Stem cell Technology NV, Noorwegenstraat 4, 9940 Evergem, Belgium; 2 Department of Medical Imaging and Orthopedics of Domestic Animals, Faculty of Veterinary Medicine, Ghent University, Salisburylaan 133, 9820 Merelbeke, Belgium; 3 Biometrics Research Center, Faculty of Veterinary Medicine, Ghent University, Salisburylaan 133, 9820 Merelbeke, Belgium

**Keywords:** Mesenchymal stem cells, Xenogeneic, Equine peripheral blood, Scintigraphy, Biodistribution, Canine

## Abstract

**Background:**

Mesenchymal stem cell treatments in dogs have been investigated as a potential innovative alternative to current conventional therapies for a variety of conditions. So far, the precise mode of action of the MSCs has yet to be determined. The aim of this study was to gain more insights into the pharmacokinetics of MSCs by evaluating their biodistribution in healthy dogs after different injection routes.

**Methods:**

Three different studies were performed in healthy dogs to evaluate the biodistribution pattern of radiolabelled equine peripheral blood-derived mesenchymal stem cells following intravenous, intramuscular and subcutaneous administration in comparison with free ^99m^Technetium. The labelling of the equine peripheral blood-derived mesenchymal stem cells was performed using stannous chloride as a reducing agent. Whole-body scans were obtained using a gamma camera during a 24-h follow-up.

**Results:**

The labelling efficiency ranged between 59.58 and 83.82%. Free ^99m^Technetium accumulation was predominantly observed in the stomach, thyroid, bladder and salivary glands, while following intravenous injection, the ^99m^Technetium-labelled equine peripheral blood-derived mesenchymal stem cells majorly accumulated in the liver throughout the follow-up period. After intramuscular and subcutaneous injection, the injected dose percentage remained very high at the injection site.

**Conclusions:**

A distinct difference was noted in the biodistribution pattern of the radiolabelled equine peripheral blood-derived mesenchymal stem cells compared to free ^99m^Technetium indicating equine peripheral blood-derived mesenchymal stem cells have a specific pharmacokinetic pattern after systemic administration in healthy dogs. Furthermore, the biodistribution pattern of the used xenogeneic equine peripheral blood-derived mesenchymal stem cells appeared to be different from previously reported experiments using different sources of mesenchymal stem cells.

## Background

Over the past decade, the use of mesenchymal stem cell (MSC) treatments in dogs has been investigated as an interesting and innovative alternative to current conventional therapies. Promising results were described for a variety of conditions such as osteoarthritis, tendon and ligament lesions, liver diseases, atopic dermatitis and inflammatory bowel disease [1–8]. The most frequently used sources of canine MSCs are autologous or allogeneic adipose tissue-derived MSCs. Other available sources include autologous synovial fluid-derived MSCs, autologous or allogeneic bone marrow-derived MSCs, allogeneic umbilical cord-derived MSCs and xenogeneic equine peripheral blood-derived MSCs [1, 3, 4]. Allogeneic and xenogeneic MSCs are a more attractive source than the autologous MSCs which have to be harvested from the tissue of each patient and put into culture before being available. Moreover, thanks to a donor selection programme based on health and quality, xenogeneic and allogeneic MSCs give the advantage to obtain a standardized ready-to-treat product. Indeed, the increase of the age of the donor is correlated to a decline in growth capacity and potency of the MSCs [9]. From a practical and economic standpoint, the use of xenogeneic MSCs is an interesting alternative since harvesting tissue from easily available healthy donor horses provides an effective technique to produce MSCs. Particularly, since MSCs are harvested from the peripheral blood, the procedure is minimally invasive and causes minimal discomfort for the animal. Additionally, by using xenogeneic MSCs, no virulent species-specific pathogens are transmitted. Finally, xenogeneic MSCs are an interesting alternative for use in dogs since canine MSCs have culture and upscaling limitations caused by senescence earlier in the culture process than for example in human and equine MSCs [10–12].

MSCs are capable of differentiating in different cell lineages, have immunomodulatory effects and stimulate local repair cells by paracrine signals [13–15]. However, additional studies are requested to further define their mode of action. The evaluation of the biodistribution of MSCs would help to gain more insights into their pharmacokinetics. Spriet et al. described the scintigraphic tracking of ^99m^Technetium-hexamethyl-propylene amine oxime (HMPAO)-labelled allogeneic adipose tissue-derived MSCs following portal, intravenous and splenic administration in four healthy dogs. To the authors’ knowledge, the study conducted by Spriet et al. is the only one describing the distribution of ^99m^Tc-labelled MSCs in dogs [16]. Scintigraphic tracking of MSCs was also reported in horses and humans [17–21].

In most of these studies, MSCs were labelled using HMPAO as a chelating agent to bind ^99m^Technetium to the MSCs [17, 19–21], and one group used a combination of HMPAO and stannous chloride for the MSC labelling [18]. Our group recently reported the labelling of equine peripheral blood-derived mesenchymal stem cells (ePB-MSCs) for scintigraphic tracking after intravenous administration in horses using stannous chloride (SnCl_2_) for the labelling of the MSCs (paper submitted). In the study reported by Spriet et al. in 2015, an intravenous regional limb perfusion and a subcutaneous injection in the metacarpal area or the coronary band were performed in horses. The subcutaneous injection resulted in a loss of the MSCs to the general circulation; however, there was no evidence of local migration [20]. To the author’s knowledge, no study has described the biodistribution of subcutaneously injected MSCs in dogs.

The aim of this study was to evaluate the biodistribution pattern of ^99m^Technetium-labelled equine xenogeneic MSCs, in comparison with free ^99m^Technetium, after intravenous (IV), intramuscular (IM) and subcutaneous (SC) application in healthy dogs.

## Methods

### Experiments

Three different studies were performed. In the first study, the biodistribution pattern of intravenously administrated ePB-MSCs was evaluated in four dogs. In the second study, the biodistribution pattern of intramuscularly and subcutaneously administrated ePB-MSCs was evaluated in four dogs. Finally, in the third study, the biodistribution pattern of a higher dose of ePB-MSCs following intravenous administration was evaluated in three dogs.

### Animals

The different animal studies (approval number EC: 2019_003 and 2019_006) and the blood collection of the donor horses (approval number: EC_2016_003) were approved by the ethics committee of Global Stem cell Technology. The ethics committee is approved by the Flemish Government with permit LA1700607. The study was good clinical practice compliant (VICH GL9), and all animal handlings were conducted according to European, national and regional animal welfare regulations (Directive 2001/82/EC as amended, Belgian Animal Welfare Legislation (KB 29/05/2013), Directive 2010/63/EU and EMEA/CVMP/816/00-Final).

Four healthy adult research dogs were included in the two first studies; three dogs from the second study were re-used for the third study. All dogs were purpose-bred adult beagles (16–23 months). Two males and two females were included in the first two studies, and one male and two female dogs were included in the third study. The dogs were housed in groups of 2, in a pen of 4 by 4 by 2 m (L × W × H), so permanent visual, olfactorial, tactile and auditive contact between dogs was possible.

A daily general physical assessment was performed for each study evaluating the following parameters: rectal temperature, respiratory rate, heart rate, mucosal membranes, capillary refill time, body conditions score, mentation and hydration.

### Control product preparation

For the preparation of the control product (CP), 20 ± 5 mCi (740 ± 185 MBq) of freshly eluted ^99m^Tc pertechnetate (^99m^Tc) from a molybdenum generator (GE Healthcare, Eindhoven, The Netherlands) was added to 1 mL of Dulbecco’s modified Eagle’s low-glucose medium (DMEM) (Life Technologies Europe BV, Belgium).

### Collection and culture of ePB-MSCs

As previously described by our group [22], the ePB-MSCs were Good Manufacturing Practices (GMP) manufactured in a GMP-certified site (number: BE/GMP/2018/123). Briefly, blood was taken from the jugular vein of a donor horse (approval number EC: EC_2012_001 and 2016_003), and the MSCs were isolated. The serum was analysed for a range of transmittable diseases by Böse laboratory (Harsum, Germany). As already described by our group [11], the blood was centrifuged and the buffy coat was collected for gradient centrifugation. After washing, the ePB-MSCs were cultivated until passage 5 and a characterization for viability, morphology, presence of cell surface markers and population doubling time was performed. Next, the ePB-MSCs were frozen as an intermediate cell stock. When characterization was completed, the intermediate cell stock was thawed and cultivated until passage 10 before being trypsinized, resuspended, filtered twice trough a 40-μm filter and vialed at 3 × 10^5^ cells/mL in a mixture of DMEM and 10% dimethylsulfoxide (DMSO). The vials were stored at − 80 °C until further use.

### ^99m^Tc-labelling of the ePB-MSCs

The technique of ^99m^Tc labelling the ePB-MSCs was based on an optimization study recently described by our group (data not shown). First, stannous chloride powder (Sigma Aldrich, US) was dissolved in sterile basic water (pH 8.5). Next, 0.9 × 10^6^ ePB-MSCs were thawed in the hand palm, transferred into a growth medium and centrifuged for pelleting. The cell pellet was then re-suspended in 4 mL of saline and mixed with 5 mg SnCl_2_ and 45 ± 5 mCi (1665 ± 185 MBq) of freshly eluted ^99m^Tc from a molybdenum generator (GE Healthcare, Eindhoven, The Netherlands). Next, the preparation was incubated for 30 min at room temperature before being centrifuged. The cell pellet was washed with 5 mL DMEM and centrifuged again. The final cell pellet was resuspended in 1 mL of DMEM, and the viability of the ePB-MSCs following the labelling was determined using trypan blue. The radioactivity of the supernatant was measured after each centrifugation step in a dosiscalibrator and used to calculate the labelling efficiency.

### Treatment

**Fig. 1:**
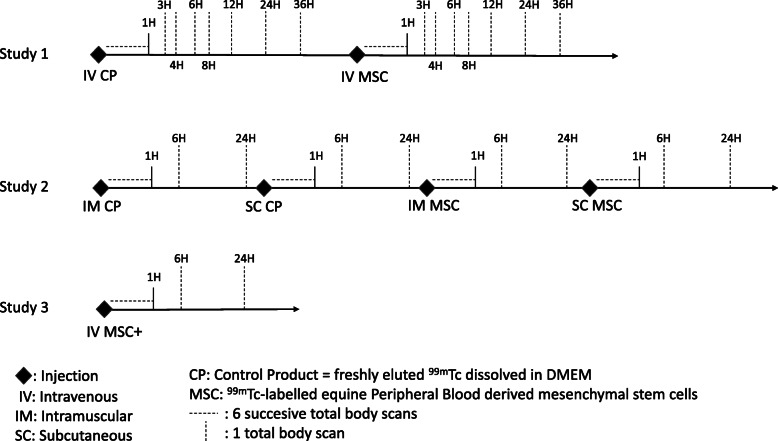
Schematic presentation of the timeline for each study

In the first study, each dog received two intravenous injections; first, the dogs were injected with the control product: freshly eluted ^99m^Tc dissolved in DMEM, and at least 7 days later, they received a second injection with ^99m^Tc-labelled ePB-MSCs. For the second study, 4 injections were administered to each dog. The dogs first received an IM and a SC injection with the control product then an IM and a SC injection with the ^99m^Tc-labelled ePB-MSCs. At least 7 days separated each injection. In the third study, the three dogs received a single IV injection with ^99m^Tc-labelled ePB-MSCs (Fig. 1).

The dogs were put under general anaesthesia and positioned in sternal recumbency on the gamma camera before each injection. To obtain general anaesthesia, the dogs were first sedated with dexmedetomidine (12–25 μg/kg IM), next induction was obtained with propofol (dosage on effect) and anaesthesia was maintained with isoflurane 1.2–1.4% (on effect) in 100% oxygen following endotracheal intubation. The intravenous injection was administered through a 22-gauge catheter in one of the cephalic veins, the intramuscular injection was performed in the left quadriceps muscle and the subcutaneous injection was administered at the back of the neck.

### Imaging protocol

A two-headed gamma camera, equipped with low-energy high-resolution collimators (GCA 7200 A; Toshiba) was used for the scintigraphic investigation. The whole-body scan was obtained with the detectors of the SPECT scanner moving simultaneously dorsally and ventrally from the head to the tail of the dog over 10 min. All dogs were kept under general anaesthesia during all the acquisitions. For all 3 studies, data collection of the first hour consisted of 6 successive acquisitions of each 10 min. The start of the first acquisition was simultaneous with the injection of the radioactive compound and the dog remained in the same position for all 6 scans. Next, in the first study, total body scans (each lasting 10 min) were performed at 3 h, 4 h, 6 h, 8 h, 12 h, 24 h and 36 h after placebo control and labelled ePB-MSC administration using propofol (dosage on effect). For the second and the third study, 6 successive 10-min total body scans performed during the first hour after injection were followed by total body scans (each lasting 10 min) at 6 h and 24 h after each injection (Fig. 1). For all studies, a syringe with a known amount of radioactivity to calculate % injected dose (ID) was simultaneously scanned with the dog. Care was taken for the dog’s re-positioning on the table, to avoid too much spatial deviation on the scans following the first hour scans.

### Image interpretation

First, the distribution of the placebo control and the labelled ePB-MSCs was assessed descriptively through the whole body. Consequently, the radioactivity was quantified in different manually drawn regions of interest (ROIs) on the dorsal and ventral view of the whole-body scans (matrix size 512 × 1024) using the free-hand region of interest tool of a DICOM viewing software platform (Hermes MultiModalityTM, Nuclear Diagnostics, Sweden). A geometric mean of dorsal and ventral activity for each time point and each ROI was calculated to compensate for attenuation. Relative uptake was expressed as percentage of decay-corrected injected activity for each region of interest per time point and calculated based on the known standard activity. To keep the shape and sizes (number of pixels) of the different organ ROIs uniform, a ROI template was created per study and per dog and used for the different time points. A specific organ ROI was drawn on the image on which the organ was best delineated and thereafter used for the other images. Due to minor positioning deviations in between scans, ROIs had to be replaced on some images, however without changing the shape and size.

Due to the low sample size of four animals, only the overall effects in the heart, lung, liver and bladder following intravenous injection in study 1 were taken into account for statistical analysis. The data were analysed with the SAS® statistical analysis software (version 9.4, SAS Institute Inc., Cary, NC, USA). For the intramuscular and subcutaneous injections, no statistical analysis was performed since a high radioactivity uptake remained at the injection sites following the injections of the radiolabelled ePB-MSCs and only a descriptive evaluation seemed appropriate. The overall statistical difference between intravenous administration of the free ^99m^Tc and the radiolabelled ePB-MSCs in the heart, lungs, liver and bladder was calculated using the area under the curve (AUC). The AUC was calculated using the trapezoidal method and can be written as a weighted sum of the observations. To allow a better interpretation of the AUC, it was presented as the weighted mean of the observations, using the weights of the observations in the AUC sum. A paired t-test was performed for this AUC for each organ separately, using the dog as a block effect. The time effects were described descriptively. The normality distribution assumption of the residuals was tested using the Shapiro-Wilks test and could not be rejected.

## Results

### Control product preparation

The injected ^99m^Tc activity for the CP injection in each dog is displayed in Table 1.

**Table 1:** ^99m^Tc activity and route of the CP injected to each dog in the different studies

Study	Dog	Injection route	Injected ^99m^Tc activity (mCi)
1	1	IV	20.04
	2		22.55
	3		22.70
	4		19.26
2	6	IM	18.60
	7		25.00
	8		22.45
	9		21.24
	6	SC	18.15
	7		21.50
	8		20.60
	9		18.96

*mCi* millicurie, *IV* intravenous, *IM* intramuscular, *SC* subcutaneous

### Labelling efficiency, post-labelling viability and injected dose

For the first study, the mean (min-max) overall labelling efficiency was 64.76% (59.58–71.50%), the number of MSCs ranged between 305,000 and 415,000 and post-labelling cell viability amounted to 84.64% (79.10–88.52%). For the IM injection of the second study, a mean overall labelling efficiency of 69.37% (53.65–77.83%), 465,000 to 775,000 MSCs and post-labelling cell viability of 93.70% (90.32–96.96%) were obtained, and for the SC administration of the same study, the mean overall labelling efficiency was 72.94% (66.21–78.34%), the number of MSCs per sample was 730,000 to 825,000 and post-labelling cell viability was 94.96% (93.63–96.96%). Finally, a mean overall labelling efficiency of 82.50% (80.65–83.82%), 1,610,000 to 1,950,000 MSCs and a cell viability of 95.40% (93.17–97.44%) were obtained in the third study (Table 2).

**Table 2:** Injection route, ^99m^Tc activity, labelling efficiency, number and viability of the labelled-ePB-MSCs injected to each dog in the different studies

Study	Dog	Injection route	Injected ^99m^Tc activity (mCi)	Labelling efficiency (%)	Number of MSCs	Viability of MSCs (%)
1	1	IV	17.36	71.50	415,000	87.95
	2		17.64	59.58	305,000	88.52
	3		19.40	67.80	385,000	83.00
	4		16.38	60.15	335,000	79.10
2	5	IM	18.45	72.78	465,000	90.32
	6		19.05	73.21	775,000	95.48
	7		24.04	77.83	695,000	93.53
	8		15.00	53.65	775,000	95.48
	5	SC	15.62	66.21	825,000	96.96
	6		20.15	69.18	785,000	93.63
	7		23.38	78.02	760,000	94.74
	8		23.49	78.34	730,000	94.52
3	6	IV	19.56	80.65	1,810,000	95.58
	7		27.85	83.04	1,950,000	97.44
	8		21.37	83.82	1,610,000	93.17

*mCi* millicurie, *IV* intravenous, *IM* intramuscular, *SC* subcutaneous

### Safety

The parameters rectal temperature, respiratory rate, heart rate, mucosal membranes, capillary refill time, body conditions score, mentation and hydration were in the physiological range for all animals at all time points of observation. No abnormal general clinical signs were observed, and no (serious) adverse events or suspected adverse drug reactions were observed during the study.

### Biodistribution

**Fig. 2:**
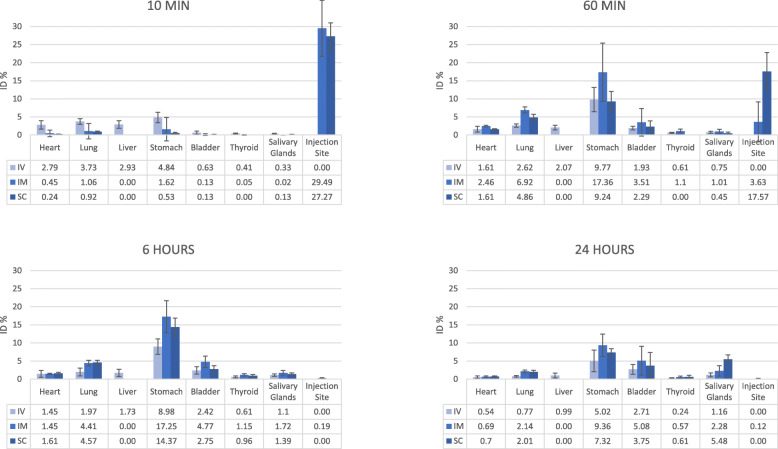
Average percentage of the injected dose observed in the different organs following the control product injections. ID, injected dose; IV, intravenous; IM, intramuscular; SC, subcutaneous

**Fig. 3:**
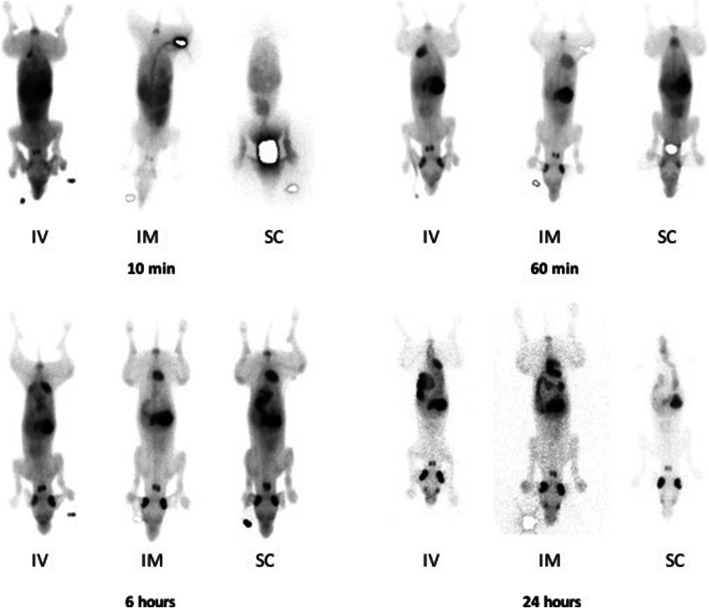
Measured radioactivity following intravenous, intramuscular and subcutaneous injection of the control product. Following intravenous (IV), intramuscular (IM) and subcutaneous (SC) administration of the in ^99m^Tc dissolved in DMEM, the heart, lung, stomach (and intestines on the later time points), thyroid, salivary glands and urinary bladder can be seen. Activity in the injection site is masked on the 10-min views of IM and SC and on the 60-min view of the SC administration

Intravenous injection of the CP led to an accumulation of free ^99m^Tc in the following organs: heart, lung, liver, stomach, bladder, thyroid and salivary glands. Following intramuscular and subcutaneous administration of the placebo control, radioactivity uptake was seen in the heart, lung, stomach, bladder, thyroid and salivary glands. The highest uptake was seen in the stomach for all three injection routes with a progressive increase until 4 to 6 h post-injection (Figs. 2 and 3). In the first study, the scintigraphic examination 36 h post-injection was performed; however, the radioactive counts were too low to quantify. Therefore, this time point was not included in the evaluation of the biodistribution pattern.

**Fig. 4:**
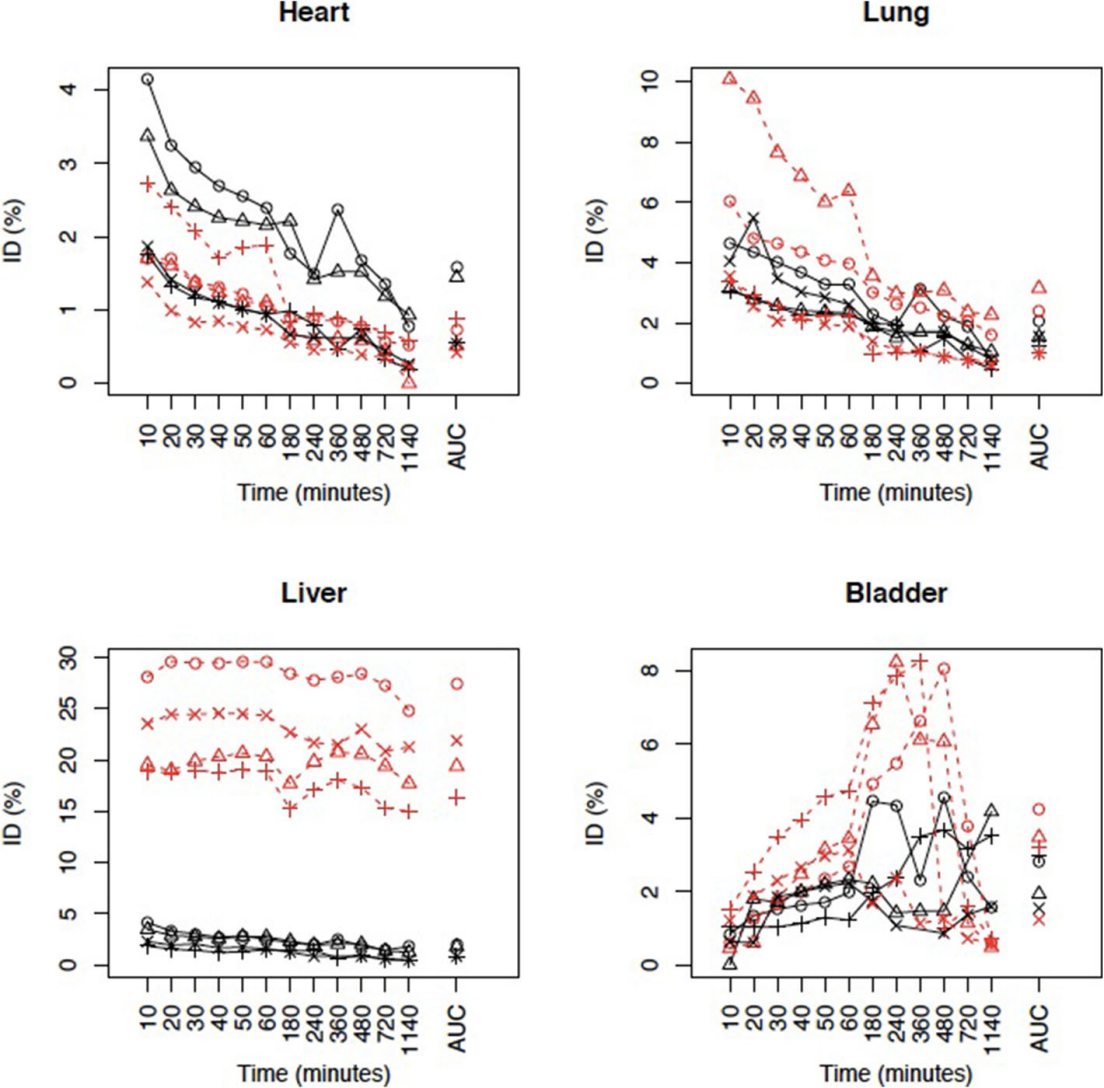
Evolution in the heart, lung, liver and bladder following intravenous injection of CP and MSCs. The evolution of time is represented for each dog in the different organs after the injection of free ^99m^Tc (black) and radiolabelled ePB-MSCs (red). A significant difference relating to the area under the curve (AUC) (P-value = 0.003) for injected dose (ID) % in the liver between the free ^99m^Tc and radiolabelled ePB-MSCs could be found following intravenous (IV) administration. No significant difference relating to the AUC was obtained following both IV injections in the heart (p = 0.28), lung (p = 0.58) or bladder (p = 0.21)

Following intravenous injections of the normal dose of the labelled ePB-MSCs, a significant difference relating to the AUC (P-value = 0.003) for ID% in the liver between the free ^99m^Tc and radiolabelled ePB-MSCs could be found. No significant difference was obtained following both IV injections in the heart (p = 0.28), lung (p = 0.58) or bladder (p = 0.21) (Fig. 4).

**Fig. 5:**
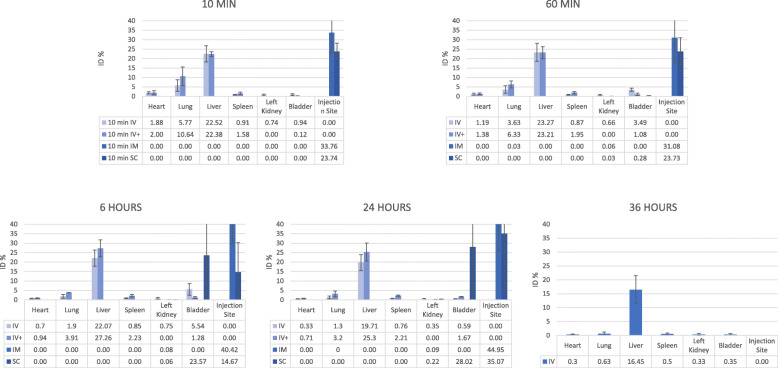
Average percentage of the injected dose observed in the different organs following the radiolabelled ePB-MSC injections. ID, injected dose; IV, intravenous (normal dose, IV; higher dose, IV+); IM, intramuscular; SC, subcutaneous

**Fig. 6:**
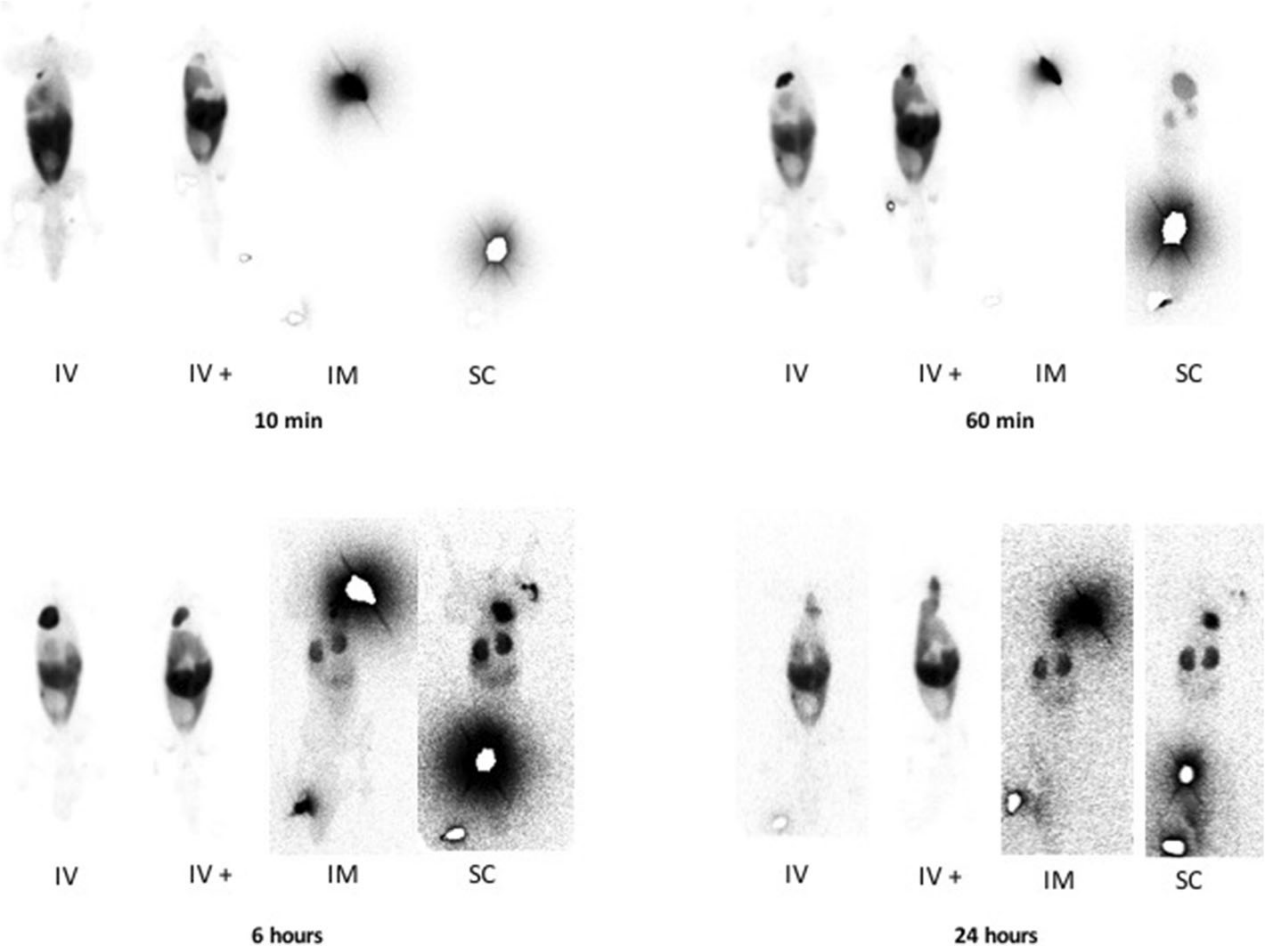
Measured radioactivity following intravenous intramuscular and subcutaneous injection of the radiolabelled ePB-MSCs. Following intravenous administration of ePB-MSCs labelled with ^99m^Tc, the heart, lung, liver, spleen, kidneys and urinary bladder can be seen. Following intramuscular injection of the ePB-MSCs radiolabelled with ^99m^Tc, the liver and kidneys can be seen. The injection site in the quadriceps muscle is clearly visible until the end of the evaluation period. Following subcutaneous injection of the ePB-MSCs radiolabelled with ^99m^Tc, the liver, kidneys and bladder can be seen. The injection site in the neck is clearly visible until the end of the evaluation period. IV, intravenous (normal dose, IV; higher dose, IV+); IM, intramuscular; SC, subcutaneous

After intravenous injections of the normal and the higher dose of the labelled ePB-MSCs into the cephalic vein of the dogs, the presence was predominantly observed in the heart, lung, liver and bladder. Furthermore, minor radioactive uptake was seen in the spleen and kidneys. The highest uptake was seen in the liver with stable radioactivity until 24 h post-injection. Intramuscular injection of the labelled ePB-MSCs led to a low radioactivity uptake in the following organs: lung, liver and kidneys. High uptake remained at the injection site for the entire evaluation period. This uptake at the injection site masked a potential uptake in the bladder. Finally, after subcutaneous injection of the labelled ePB-MSCs, low radioactivity uptake in the following organs was seen: kidneys and bladder. Again, high uptake remained at the injection site for the entire evaluation period. Radioactive uptake could be seen in the liver; however, this uptake was too low to be quantified. The uptake at the injection site masked a potential uptake in the heart and/or lung (Figs. 5 and 6).

## Discussion

The different studies describe the total body distribution pattern of intravenously, intramuscularly and subcutaneously injected ^99m^Tc-labelled ePB-MSCs compared with free ^99m^Tc during a 24-h follow-up period with scintigraphy in healthy dogs. To the authors’ knowledge, this is the first study comparing the biodistribution pattern of ^99m^Tc-labelled xenogeneic ePB-MSCs with free ^99m^Tc in dogs. Furthermore, total body scintigraphy after intravenous, intramuscular and subcutaneous injection of ^99m^Tc-labelled ePB-MSCs has never been described.

The labelling efficiency and cell viability ranged between 59.58 and 83.82% and between 79.10 and 97.44% for all studies, respectively. This is considerably higher than the labelling efficiency of 42 to 57% reported by Spriet et al. where the MSCs were labelled with ^99m^Tc-HMPAO. However, no post-labelling cell viability was reported for this study [16].

Free ^99m^Tc is preferentially taken up by the stomach, thyroid gland and salivary glands [23]. This was also seen in the current studies for the different injection routes (i.e. IM, SC and IV). No radioactive accumulation was observed in none of these organs at all time points following the different injection routes of ^99m^Tc-labelled ePB-MSCs. Therefore, we could confirm the used ^99m^Tc labelling technique resulted in a stable in vivo complex with ePB-MSCs. Additionally, the labelling did not affect the viability of the ePB-MSCs after injection, since it is assumed that cell death would cause a release of ^99m^Tc since the cell membrane is no longer intact and accumulate in the aforementioned organs similar to free ^99m^Tc injections. A lower uptake of free ^99m^Tc was seen in the heart and lung following all injections and in the liver after intravenous injection. Finally, the previously described partial excretion route of ^99m^Tc through glomerular filtration explains the increased uptake in the bladder [23].

In contrast to the observations reported by Spriet et al., a high liver uptake was seen following both intravenous injections (i.e. study 1 and study 3) of the ePB-MSCs which remained stable during the first 6 h following the injection and only decreased slightly 24 h post-injection [16]. A significant difference between the free ^99m^Tc and radiolabelled ePB-MSCs relating to the AUC (P-value = 0.003) for ID% in the liver was seen for study 1. These findings support the potential use of intravenously administered ePB-MSCs for the treatment of liver diseases such as acute liver injuries or hepatocutaneous syndrome [6, 8].

No pronounced initial pulmonary trapping of the ePB-MSCs following the different injection routes was seen. In the third study, more ID % was detected in the lungs; however, this can be explained by the higher amount of cells injected. There was initial accumulation in the lungs after injecting the higher dose, indicating no long-term entrapment of the ePB-MSCs occurs after IV injection. In contrast, other groups using technetium-labelled mesenchymal stem cells described initial high pulmonary trapping [16, 24]. The absence of pulmonary entrapment in the current studies could be explained by the use of a different MSC source and a lower number of injected MSCs. In the study reported by Spriet et al., 10 × 10^6^ adipose tissue-derived MSCs were injected in the same dog breed as in our studies, whereas our group injected only 305,000 to 1,950,000 equine peripheral blood-derived MSCs in the dogs. Moreover, a part of the production process of the ePB-MSCs used in this study consists of a filtration process which might have reduced the risk of cell clustering following the intravenous injection of the ePB-MSCs.

Following IM and SC injection, only a very low distribution of the radiolabelled ePB-MSCs was seen, and a high amount of the injected MSCs stayed at the injection site throughout the 24-h follow-up period. Consequently, the biodistribution pattern of the ePB-MSCs following IM and SC injections appears to be different from intravenously injected ePB-MSCs.

The limitations of the studies were the absence of blinding and randomization. Blinding was not feasible because the washout period of the radiolabelled ePB-MSCs is currently unknown and therefore could not be administered first before the free ^99m^Tc. This practical constraint together with the reported knowledge on distribution of free ^99m^Tc in the literature [23] would have meant that the investigator could have guessed with high certainty which animals would have received the free ^99m^Tc and which ones the radiolabelled ePB-MSCs when evaluating the total body scans. However, the absence of blinding was mitigated by using an objective evaluation criterion for evaluation of biodistribution, i.e. scintigraphic total body scans for quantifying radioactivity in a region of interest instead of using subjective scores. Another limitation was the low number of dogs included in the studies which limited the possibilities for statistical analysis. Except for the heart, lung, liver and bladder following intravenous injection in study 1, the other results were not taken into account for statistical analysis. The other results are not based on statistical tests and can therefore only be considered exploratory. In addition, due to the short half-life of ^99m^Tc, the distribution of the MSCs could only be evaluated for 24 to 36 h. Finally, although no abnormal general clinical signs were observed and no (serious) adverse events or suspected adverse drug reactions were seen in the dogs, because of the short study period, the long-term effect of repeated injections of xenogeneic MSCs in dogs is unknown. Additional studies evaluating the long-term safety of repeated injections of xenogeneic MSCs dogs will need to be performed.

## Conclusions

This study describes the biodistribution pattern of radiolabelled ePB-MSCs following intravenous, intramuscular and subcutaneous injection in dogs measured by scintigraphic evaluation of radioactivity. During this study, a distinct difference was noted in the biodistribution pattern of the radiolabelled ePB-MSCs and free ^99m^Tc. This implies ePB-MSCs have a specific pharmacokinetic pattern after systemic administration in healthy animals. This study thus gives indications for more targeted sampling during safety studies. Additionally, it also provided information on the biodistribution pattern of the used xenogeneic ePB-MSCs which appeared to be different from previously reported experiments using different MSC sources.

## Data Availability

The datasets used and/or analysed during the current study are available from the corresponding author on reasonable request.

## Abbreviations

MSC: Mesenchymal stem cell
HMPAO: Hexamethyl-propylene amine oxime
ePB-MSCs: Equine peripheral blood-derived mesenchymal stem cells
SnCl2: Stannous chloride
IV: Intravenous
IM: Intramuscular
SC: Subcutaneous
^99m^Tc: ^99m^Tc pertechnetate
DMEM: Dulbecco’s modified Eagle’s low-glucose medium
GMP: Good Manufacturing Practices
DMSO: Dimethylsulfoxide
ID: Injected dose
ROIs: Regions of interest
AUC: Area under the curve
